# Emerging azithromycin-resistance among the *Neisseria gonorrhoeae* strains isolated in Hungary

**DOI:** 10.1186/s12941-016-0166-9

**Published:** 2016-09-20

**Authors:** Alexandra Brunner, Eva Nemes-Nikodem, Csaba Jeney, Dora Szabo, Marta Marschalko, Sarolta Karpati, Eszter Ostorhazi

**Affiliations:** 1 Department of Dermatology, Venerology and Dermatooncology, Semmelweis University, 41 Mária Street, Budapest, Hungary; 2 Department of Laboratory Medicine, Semmelweis University, 41 Mária Street, Budapest, Hungary; 3 Institute of Medical Microbiology, Semmelweis University, 4 Nagyvárad Square, Budapest, Hungary

**Keywords:** Azithromycin-resistance, *Neisseria gonorrhoeae*, Sequence types, Phylogenetic tree

## Abstract

**Background:**

In the 1990s, azithromycin became the drug of choice for many infectious diseases but emerging resistance to the drug has only been reported in the last decade. In the last 5 years, the National *Neisseria gonorrhoeae* Reference Laboratory of Hungary (NNGRLH) has also observed an increased number of *N. gonorrhoeae* strains resistant to azithromycin. The aim of this study was to determine the most frequent sequence types (ST) of *N. gonorrhoeae* related to elevated levels of azithromycin MIC (minimal inhibitory concentration). Previously and currently isolated azithromycin-resistant strains have been investigated for the existence of molecular relationship.

**Methods:**

Maldi-Tof technic was applied for the identification of the strains isolated from outpatients attending the reference laboratory. Testing antibiotic susceptibility of azithromycin, cefixime, ceftriaxone, tetracycline, spectinomycin and ciprofloxacin was carried out for all the identified strains, using MIC strip test Liofilchem^®^. *N. gonorrhoeae* multiantigen sequence typing (NG-MAST) was performed exclusively on azithromycin-resistant isolates. A phylogenetic tree was drawn using MEGA6 (Molecular Evolutionary Genetics Analysis Version 6.0) Neighbour-Joining method.

**Results:**

Out of 192 *N. gonorrhoeae* isolates, 30.0 % (58/192) proved resistant to azithromycin (MIC > 0.5 mg/L). Of the azithromycin-resistant isolates, ST1407, ST4995 and ST11064 were the most prevalent. Based on the phylogenetic analysis, the latter two STs are closely related.

**Conclusions:**

In contrast to West-European countries, in our region, resistance to azithromycin has increased up to 30 % in the last 5 years, so the recommendation of the European Guideline −500 mg of ceftriaxone combined with 2 g of azithromycin as first choice therapy against *N. gonorrhoeae*- should be seriously considered in case of Hungary.

**Electronic supplementary material:**

The online version of this article (doi:10.1186/s12941-016-0166-9) contains supplementary material, which is available to authorized users.

## Background

The treatment of gonorrhoea infection poses a continuous problem as *Neisseria gonorrhoeae* has developed resistance to each antimicrobials used in the past 70 years [1]. Therefore, it is necessary to enhance the surveillance of gonococcal antimicrobial resistance, especially for the drugs of first choice: ceftriaxone and azithromycin [2]. In Hungary, resistance to ceftriaxone has not yet been reported. In contrast, the appearance and spread of azithromycin-resistance have been observed in the last 4 years [3].

Since the 1990s, azithromycin has become the drug of choice for many infections, such as sexually transmitted diseases (STDs), community-acquired pneumonia, acute bacterial sinusitis, otitis media, tonsillitis, pharyngitis, skin infections or acute bacterial exacerbations of chronic obstructive pulmonary disease [4]. Of STDs, azithromycin is used to treat uncomplicated gonorrhoea in patients with cephalosporin allergy, *Chlamydia trachomatis* co-infection, *Heamophilus ducreyii, Ureaplasma urealyticum, Mycoplasma genitalium* infections. This antibiotic revolutionised the therapy as it shortened treatment time from 7–14 days to 1–5 days and improved patient compliance due to high tissue levels and long half-life. New administration formulations such as sustained-release microspheres allowed higher doses to be administered and reduced gastrointestinal side-effects, so azithromycin seemed to be capable of approaching the concept of an ideal antibiotic [5]. However, recently decreased antimicrobial susceptibility to azithromycin may disprove this assumption.

According to the data of the European Surveillance of Antimicrobial Consumption (ESAC), in Hungary the outpatient consumption of antimicrobials was 16.0 defined daily doses (DDD) per 1000 inhabitants per day. This number can be subdivided into major antibiotic classes such as penicillins topping the list by DDD of 7.19, macrolides taking the second place with DDD of 2.94 and, finally, cephalosporins with DDD of 2.13 [6].

Nevertheless, at the National *Neisseria gonorrhoeae* Reference Laboratory of Hungary (NNGRLH), we observed the appearance and rapid spread of azithromycin-resistance in Hungary between 2010 and 2013. We aimed to survey the antimicrobial susceptibility in 2014 and compare it with the data of the last 4 years and characterise the azithromycin-resistant strains by NG-MAST.

Molecular evolutionary analysis was conducted and genetic relationships were estimated between the STs spreading in Hungary in 2014.

## Methods

### Bacterial strains and medical records

The NNGRLH at the STD Centre in the Department of Dermatology, Venerology and Dermatooncology of Semmelweis University, Budapest, Hungary collected samples from consecutive symptomatic gonorrhoea patients and from their asymptomatic contacts in 2014. The samples were cultured, characterised and stored on Cryobank breads (Mast Diagnostic, Germany) at −80 °C. Clinical data such as sex, age, sexual orientation, anatomic site of infection were recorded. *C. trachomatis* co-infection was also screened. Patients’ data were analysed according to law 1997/CLIV 26§ taking into account maximum privacy rights and anonymity of patients [7].

### Antibiotic susceptibility

Clinical samples -cervical, anal, urethral and pharyngeal swabs- were obtained and grown on preheated VCA3 agar (Biomérieux, Budapest, Hungary) and on non-selective PVX chocolate agar (Biomérieux, Budapest, Hungary) at 37 °C in an atmosphere of 5 % of carbon dioxide for 24–48 h. Minimum inhibitory concentrations (MIC; mg/L) were determined for azithromycin, cefixime, ceftriaxone, tetracycline, spectinomycin and ciprofloxacin on PVX chocolate agar (Biomérieux, Budapest, Hungary) using MIC strip tests (Liofilchem^®^ s.r.l., Roseto degli Abruzzi, Italy) according to the manufacturer’s instructions, using a direct colony suspension equivalent to McFarland standard of 0.5. Testing conditions also included incubation at 36.5 °C and 5 % of carbon dioxide for 24 h. All results were interpreted by using breakpoints for susceptibility and resistance according to the European Committee on Antimicrobial Susceptibility Testing (EUCAST) [8]. Concerning the MIC breakpoints of azithromycin, strains with MICs over 0.25 mg/L but below 0.5 mg/L were considered to be of intermediate resistance. Isolates with MICs higher than 0.5 mg/L were considered resistant. *N. gonorrhoeae* ATCC 49226, with an azithromycin MIC of 0.12 mg/L, was used as a control strain to ensure the quality of the susceptibility tests.

### Molecular methods

Out of the 58 resistant and 42 intermediately resistant strains 29 and 21 were selected for *N. gonorrhoeae* multiantigen sequence typing (NG-MAST) according to a previously described method [9]. The sequences of *porB* and *tbpB* PCR products were determined after their preliminary purification by the Exosap IT purification kit (Affymetrix, USA). BigDye^®^ Terminator v3.1 Cycle Sequencing Kit (Life Technologies, USA) was used and the same forward and reverse primers were applied as for *porB* and *tbpB* PCR methods. Last purification was carried out by NucleoSEQ Column PCR Purification Kit (Macherey–Nagel, Germany). Nucleotide sequences were determined by capillary electrophoresis, with a capillary length of 50 cm and POP-7 polymer on ABI 3130xl Genetic Analyzer (Applied Biosystems, Foster City, CA, USA).

NG-MAST STs including the new alleles and STs were assigned on the NG-MAST website (www.ng-mast.net).

Phylogenetic tree was constructed by MEGA6 (Molecular Evolutionary Genetics Analysis Version 6.0) Neighbour-Joining algorithm, using maximum composite likelihood model [10]. The degree of similarity was determined using the highly similar sequence (Megablast) BLASTN Program of the National Library of Medicine of the National Center for Biotechnology Information [11].

## Results

In 2014, 192 *N. gonorrhoeae* strains were isolated at the STD Centre of the Department of Dermatology, Venerology and Dermatooncology of Semmelweis University, Budapest, Hungary. The number of patients attending our STD centre makes up about 10 % of the total number of notified gonorrhoea infections in Hungary year by year. However, the ratio of *N. gonorrhoeae* positive patients to total patients examined increased from 7.8 % in 2013 to 10.85 % by the end of 2014. Of the 192 *N. gonorrhoeae,* 85 % were isolated from male patients (median age 32 years); the remaining strains were collected from females (median age 26 years).

Urethritis was found in 77.3 % of male patients, while in females the dominant anatomical site of infection was the urethra (68.9 %) and cervix (65.5 %). Symptomatic infections or asymptomatic carrier states were detected in the anus (20.2 %/44.8 %) and in the pharynx (17.9 %/24.1 %) in male/female patients, respectively.

All the 192 isolates were susceptible to ceftriaxone and spectinomycin. The prevalence of ciprofloxacin and tetracycline resistance −39.8 and 70 %, respectively- remained as high as in previous years in Hungary. However, the MIC averages of ceftriaxone and cefixime have increased in the last few years. Cefixime MIC exceeded the resistant breakpoints in 1.57 % of the strains.

Of the 192 strains, 92 (48 %) were susceptible to azithromycin and 100 (52 %) exhibited reduced susceptibility. Fifty-eight of these 100 strains −30.0 % of all the strains- were resistant to azithromycin, according to the breakpoints of EUCAST. The percentage of azithromycin resistance showed a significant increase from 15.9 % in 2013 to 30.0 % in 2014 (χ^2^ = 11.4437, *P* value is 0.000717, *P* < 0.001). Concerning the strains with reduced susceptibility to azithromycin, we can say that the ratio of female/male patients was 1–7.3. MICs of ≥1 mg/L for azithromycin were observed in 7.0 % (13/192) of the isolated *N. gonorrhoeae* strains out of which three had an MIC of 1.5 mg/L (Additional file 1: Figure S1).

The prevalence of *C. trachomatis* infection detected by multiplex RT-PCR was only 6.7 % in 2014 at the NNGRLH, but *N. gonorrhoeae* positivity was found in 12.2 % of cases. Only 14.7 % of the gonorrhoea-positive samples were co-infected with *C. trachomatis*.

The 50 *N. gonorrhoeae* resistant or intermediate-resistant isolates to azithromycin were divided into 34 NG-MAST sequence types, and a unique NG-MAST sequence type was found for 10 isolates. The three dominant strains were ST1407, ST4995 and ST11064, each represented by 5 isolates (10–10 %). Regarding frequency, these were followed by ST 4417 represented by 3 isolates, then by ST 995 and ST 8517, each represented by 2 isolates. The 29 other STs were represented by only one isolate. Ten new STs, which had not been previously described in the world, were assigned as ST 11699 to ST 11708 on NG-MAST website. Four of them, ST 11703, 11706-11708, due to new allele combinations of known *porB* and *tbpB* alleles, were assigned on the website. The other 6 new STs had new *porB* or *tbpB* alleles (Table 1).

**Table 1:** Incidence of sequence types, *porB* and *tbpB* allels among azithromycin-resistant or intermediate-resistant *N. gonorrhoeae* strains isolated in Hungary in 2014

ST	*porB* allele	*tbpB* allele	Number of strains	Azithromycin susceptibility category, number of strains	
				I	R
10081	5921	29	1	1	–
10083	3031	29	1	1	–
995	28	29	2	–	2
4417	2707	894	3	1	2
10087	35	29	1	–	1
5333	3229	137	1	–	1
225	4	4	1	–	1
10088	2700	4	1	1	–
11706	1183	1388	1	–	1
7232	1489	1388	1	–	1
11702	6870	1582	1	1	–
8706	35	1582	1	1	–
11704	6871	2003	1	–	1
11708	1183	18	1	–	1
8465	4864	18	1	–	1
2400	1489	563	1	–	1
8115	3942	563	1	–	1
11707	4864	563	1	–	1
10101	4160	110	1	1	–
8517	1142	1531	2	1	1
3378	2043	110	1	1	–
8826	5213	110	1	–	1
1407	908	110	5	2	3
11699	6867	138	1	1	–
21	14	33	1	–	1
11064	14	1131	5	2	3
11703	1582	1131	1	1	–
10593	581	1131	1	1	–
11700	6868	1131	1	1	–
4995	3031	33	5	3	2
5343	6195	1131	1	–	1
11337	6630	1131	1	–	1
11705	6872	2004	1	1	–
11701	6869	1131	1	–	1
∑=			50	21	29

**Fig. 1:**
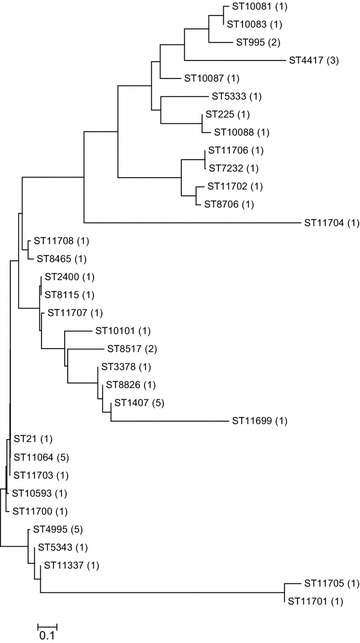
Phylogenic tree constructed from *porB* and *tbpB* allels (ST) from 50 azithromycin-resistant or intermediate-resistant strains isolated in 2014 in Hungary. Number of isolates according to the appropriate STs is indicated in parenthesis

According to the phylogenic tree in Fig. 1, the azithromycin-resistant and intermediately resistant strains isolated in NNGLRH in 2014 were divided into three major groups based on closer relationship. From the most prevalent STs, the first group contained ST225; ST1407 belonged to the second group, whereas the third group contained ST4995 and ST11064. A similarity of at least 96 % can be shown for all members of the third group. The biggest similarity-99 %- was detected between ST21, ST11064 and ST11703. A 98 % similarity between ST11703 and ST10593, or between ST10593 and ST11700 was detected, but ST11700 and ST11703 demonstrated only a 97 % similarity. With the latter two STs, ST4995 showed a similarity of 96 %. Between ST 4995 and ST5343 or ST11337 a similarity of 97 % was detected.

## Discussion

For the treatment of gonorrhoea, the European Guideline, published in 2012, recommends 500 mg of ceftriaxone combined with 2 g of azithromycin as first choice therapy [2]. This dual therapy is supported by European epidemiological surveys, mostly based on western European data. These European epidemiological surveys demonstrate that *C. trachomatis* co-infection is so common in young heterosexual patients under 30 and in MSM with gonorrhoea that 1 g of azithromycin administered orally as a single dose or 100 mg of doxycycline administered orally twice daily for 7 days should be given unless co-infection has been excluded by NAAT testing.

Since resistance to azithromycin increased from 0 to 30 % in Hungary in the last 5 years, and WHO recommended [1] that an antimicrobial should not be used when >5 % of the strains are resistant, we aimed to conduct an active surveillance to detect recent emergence in Hungary. Our theory for the increasing azithromycin resistance is that, according to ESAC, azithromycin has become the second most commonly used antimicrobial in Hungary. Hence, patients might have been treated previously with azithromycin for an infection, microbiome in the pharynx or anus, could have been exposed to this antibiotic several times, could have acquired resistance and acted as reservoirs of genes [12]. Azithromycin-resistant samples were frequently isolated from the pharynx (21 %) or the anus (32.5 %), which may refer to the fact that the asymptomatic carrier state provides an opportunity for commensal microbiome and *N. gonorrhoeae* to exchange their resistance genes, resulting in a hyperexpression of the efflux pump MtrCDE or mutation in the 23S rRNA [13, 14]. The high percentage of infections in the pharynx and anus do not only involve the risk of acquiring resistance, but also the risk of developing a disseminated infection and the spread of asymptomatic gonorrhoea.

Previously, the most frequent isolated STs were ST2992, ST1407, ST4995 and ST225 in Hungary [15]. In our recent study, three groups of STs based on closer relationship, were observed among azithromycin-resistant or intermediate-resistant *N. gonorrhoeae* strains isolated in Hungary in 2014. The first group contains the previously described ST225; ST1407 belongs to the second group, while ST4995 and ST11064 are included in the third group (Fig. 1). The latter two STs represent the 20 % of the azithromycin-resistant strains but, according to the data of Table 1 and Fig. 1, it can be presumed that more than 30 % of the azithromycin-resistant isolates are closely related in the third group of STs (similarity is at least 96 %). The previously described uniquely high prevalence of ST4995 in Hungary confirms the theory that the isolates of the third neighbourhood group are successful strains in this country, and may cause therapeutic failure in our region in the future. Nevertheless, based on the exact definition of genogroup—one identical allele is shared and the other allele shows a similarity of ≥99 % [16]—only ST21, ST11064 and ST11703 make up a genogroup. This genogroup is named G11064, since ST11064 is the predominant ST within the group.

The other prevalently isolated strains in the second group of neighbourhood are associated with ST1407, i.e. the ST previously described as resistant to ciprofloxacin, tetracycline and strongly associated with decreased susceptibility to cephalosporins [16, 17]. Poorly controlled use of antibiotics may promote the selection and spread of multidrug-resistant strains from this group [18].

The rapid selection of azithromycin-resistant strains in Hungary shows that azithromycin might not be optimised for the treatment of gonorrhoea, neither in monotherapy nor in dual therapy. On the one hand, the question is whether the combination of cephalosporins with azithromycin decreases the MICs of cephalosporins or not. There are some studies which report in vitro synergy between third-generation cephalosporins and azithromycin [19], but others do not [20]. The clinical efficacy of the dual therapy with ceftriaxone and azithromycin could be lower than ceftriaxone monotherapy since the combination was less bactericidal as ceftriaxone alone in a time-kill experiment [21].

On the other hand, we have to investigate the prevalence of *C. trachomatis* co-infection. In Europe, according to ECDC’s data, *Chlamydia* is the most frequently reported STD. In 2011, 346911 cases were notified in 25 European countries, and 39179 cases of gonorrhoea were registered. By contrast, in our laboratory twice as many gonorrhoea infections were identified as *Chlamydia* infections; 6.7 % of the samples of the STD centre were *Chlamydia*-positive and 12.2 % were gonorrhoea-positive. These data correlate with those of the National Epidemiological Laboratory, where 1077 cases of *Chlamydia* and 1525 cases of gonorrhoea were recorded in 2013. While the surveys of IUSTI refer to the common prevalence of co-infection, in our laboratory only 14.7 % of the gonorrhoea-positive samples were co-infected with *C. trachomatis*. This also raises the question whether combination therapy is the appropriate treatment in Hungary for gonorrhoea infection. Furthermore, as the increasing azithromycin-resistance and also the emerging cefixime and ceftriaxone MICs threaten the currently recommended therapies for gonorrhoea, it would be essential to replace these antimicrobials with novel ones which have not been used before for gonorrhoea infection in our country. The use of solithromycin, gentamicin, gemifloxacin might be a short term solution, but developing novel antimicrobials is essential [22, 23].

## Conclusions

In Hungary, the treatment of gonorrhoea infections relies on the combination therapy of ceftriaxone and azithromycin, recommended by the international guidelines. However, the data of this study should not only draw attention to caution in the use of azithromycin as the sole treatment for gonorrhoea but also to that in the use of combination therapy in Hungary. This study indicates that the rate of azithromycin-resistant strains is growing in our country year by year. We observed a unique emergence of azithromycin-resistant *N. gonorrhoeae* strains the amount of which has doubled in a period of 1e year. Besides, the strains being the most common in Hungary in 2013, also appeared in 2014 and were associated with azithromycin-resistance. This experience restricts the usefulness of this antibiotic recommended as first-line treatment for gonorrhoea worldwide and argues for regular surveillance to determine azithromycin-susceptibility.

## Additional file

10.1186/s12941-016-0166-9 Distribution of azithromycin MIC among *N. gonorrhoeae* strains in 2014.

## Abbreviations

ECDC: European Centre for Disease Prevention and Control
ESAC: European Surveillance of Antimicrobial Consumption
EUCAST: European Committee on Antimicrobial Susceptibility Testing
DDD: defined daily doses
IUSTI: International Union against Sexually Transmitted Infections
NG-MAST: *N. gonorrhoeae* multiantigen sequence typing
MEGA6: Molecular Evolutionary Genetics Analysis Version 6.0
MIC: minimal inhibitory concentration
MSM: men who have sex with men
NNGRL: National *Neisseria gonorrhoeae* Reference Laboratory of Hungary
ST: sequence type
STD: sexually transmitted diseases
