# Supplementary figures and images for: Emerging azithromycin-resistance among the Neisseria gonorrhoeae strains isolated in Hungary
Source: Ann Clin Microbiol Antimicrob. 2016 Sep 20;15:53. doi: 10.1186/s12941-016-0166-9 (PMC5029006; doi:10.1186/s12941-016-0166-9)

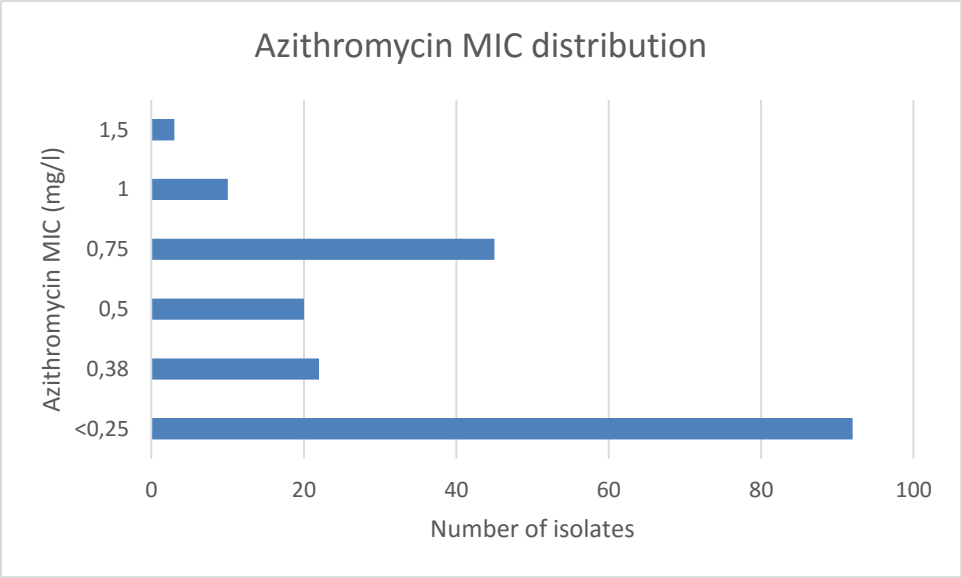

Supplement: Supplementary file 1 — 10.1186/s12941-016-0166-9 Distribution of azithromycin MIC among N. gonorrhoeae strains in 2014. [file 12941_2016_166_MOESM1_ESM.pdf]
